# p53 and rapamycin are additive

**DOI:** 10.18632/oncotarget.4602

**Published:** 2015-06-23

**Authors:** Barbara Christy, Marco Demaria, Judith Campisi, Jing Huang, Diane Jones, Sherry G. Dodds, Charnae Williams, Gene Hubbard, Carolina B. Livi, Xiaoli Gao, Susan Weintraub, Tyler Curiel, Z. Dave Sharp, Paul Hasty

**Affiliations:** ^1^ Departments of Molecular Medicine and Institute of Biotechnology, University of Texas Health Science Center at San Antonio, San Antonio, TX, USA; ^2^ Department of Pathology, University of Texas Health Science Center at San Antonio, San Antonio, TX, USA; ^3^ Department of Biochemistry, University of Texas Health Science Center at San Antonio, San Antonio, TX, USA; ^4^ Department of Medicine, University of Texas Health Science Center at San Antonio, San Antonio, TX, USA; ^5^ Cancer Therapy & Research Center, University of Texas Health Science Center at San Antonio, San Antonio, TX, USA; ^6^ Barshop Institute for Longevity and Aging Studies, University of Texas Health Science Center at San Antonio, San Antonio, TX, USA; ^7^ Buck Institute for Research on Aging, Novato, CA, USA; ^8^ Center for Cancer Research, National Cancer Institute, Bethesda, MD, USA; ^9^ Current address: Agilent Technologies, Inc., Santa Clara, CA, USA

**Keywords:** mTOR, p53, rapamycin, longevity, SASP

## Abstract

Mechanistic target of rapamycin (mTOR) is a kinase found in a complex (mTORC1) that enables macromolecular synthesis and cell growth and is implicated in cancer etiology. The rapamycin-FK506 binding protein 12 (FKBP12) complex allosterically inhibits mTORC1. In response to stress, p53 inhibits mTORC1 through a separate pathway involving cell signaling and amino acid sensing. Thus, these different mechanisms could be additive. Here we show that p53 improved the ability of rapamycin to: 1) extend mouse life span, 2) suppress ionizing radiation (IR)-induced senescence-associated secretory phenotype (SASP) and 3) increase the levels of amino acids and citric acid in mouse embryonic stem (ES) cells. This additive effect could have implications for cancer treatment since rapamycin and p53 are anti-oncogenic.

## INTRODUCTION

Rapamycin is a naturally occurring antifungal compound produced by bacteria. It forms a complex with FKBP12 that allosterically inhibits primarily mTORC1 [1, 2]. mTOR is a serine/threonine kinase found in two complexes, mTORC1 and mTORC2. mTORC1 coordinates cell growth and metabolism in response to environmental stresses, nutrient and energy levels, growth factors and other conditions [3]. Rapamycin extends life span of wild type mice [4], possibly through cancer suppression since mTORC1 signaling is often dysregulated in cancer cells [5]. In addition, rapamycin reduced cancer in kidney transplant patients [6, 7] and mouse cancer models [8-13]. Dietary rapamycin also ameliorated general aging in wild type mice for most [4, 14-17], but not all studies [18]. Rapamycin also extended life span for lower organisms that do not die from cancer suggesting that rapamycin suppresses general aging in addition to cancer [19]. Thus, rapamycin appears to suppress mTORC1-mediated oncogenesis and possibly general aging.

*p53* is a transcription factor with broad biological function [20] that is best known for suppressing tumors in humans [21, 22] and mice [23, 24] by inducing cell cycle arrest, apoptosis and senescence in response to a variety of stresses [25]. In response to stress, p53 inhibits mTORC1 [26, 27] by inducing transcription of Sestrin 1 and Sestrin 2 that activates AMP-responsive protein kinase (AMPK) to phosphorylate the mTOR inhibitor, tuberous sclerosis 2 (TSC2) [28] and by negatively regulating the amino acid-sensing pathway through modulation of Gator2 [29] or as RagA/B guanine nucleotide dissociation inhibitors (GDIs) [30]. p53 also induced PTEN/PIP3/AKT to decease mTORC1 activity [31]. Thus, p53 suppresses mTORC1-driven cell growth in response to cellular stresses like DNA damage.

Rapamycin could have an additive effect with p53 to suppress mTORC1. This possibility is especially important since most cancers are dysfunctional for p53 [32]. In support, p53-mutant mice exhibited increased mTOR activity in some, but not all tissues [33]. Yet, rapamycin extended the life span of *p53^+/−^* [10] and *p53^−/−^* [11] mice when added to the drinking water or administered in nanoformulated micelles (Rapatar), respectively. These latter results are inconsistent with the notion that p53 regulates mTORC1. However, the impact p53 dose has on rapamycin activity was not assessed in these studies. p53 dose could be important since *p53^+/−^* mice exhibit haploinsufficiency as demonstrated by tumors that maintained wild type p53 function [34]. Furthermore, increasing the dose of enterically encapsulated rapamycin (eRapa) from 14 ppm to 42 ppm proportionately enhanced mouse life span [35] and suppressed intestinal adenomas [9]. Thus, the pharmacological concentration of rapamycin could be important with regard to p53 gene dose or p53 activity.

In this study, we report three observations that support an additive relationship between p53 and rapamycin in mammals. First, p53 enabled rapamycin-driven life span extension in mice. Second, p53 facilitated rapamycin-mediated SASP reduction in human cells that were made senescent by IR. Third, p53 enhanced rapamycin-induced elevation of amino acids and citric acid in mouse embryonic stem (ES) cells. Thus, p53 augments rapamycin.

## RESULTS

### p53 augments rapamycin-induced lifespan extension in mice

We tested the possibility that p53 and rapamycin would have an additive effect at influencing mouse life span since both suppress tumors to extend life span. Mice with varying p53 levels (*p53^−/−^*, *p53^+/−^*, *p53^+/+^*) were generated using *p53^+/−^* breeding pairs (129XC57Bl-6J) [23]. Same-sex littermates were housed together (5 per cage) independent of genotype. Mice were fed chow containing empty Eudragit capsules (control) or capsules containing 14 ppm eRapa, the concentration previously shown to extend lifespan, delay tumor-related death, retard a number of age-related pathologies, and improve cerebral function [4, 8, 9, 14, 16, 17, 36]. These treatments were started at approximately 8.5 weeks of age (median age 61 days) and continued until death. Mice either died naturally or were sacrificed when moribund (unresponsive, dehydrated and immobile). At the time of death, the serum rapamycin concentration was greater in eRapa-fed mice (range 0.59 – 7.13 ng/ml, mean 2.57 +/− 1.98 ng/ml, 25 mice observed) than control-fed mice (range 1 – 1.25 ng/ml, mean 1.19 +/− 0.11 ng/ml, 17 mice observed). Most likely some eRapa-fed mice had low serum rapamycin concentrations because they had stopped eating due to their moribund condition and since blood levels were higher in healthy mice fed 14 ppm eRapa in a previous study (32 - 51 ng/ml) [9].

**Figure 1:**
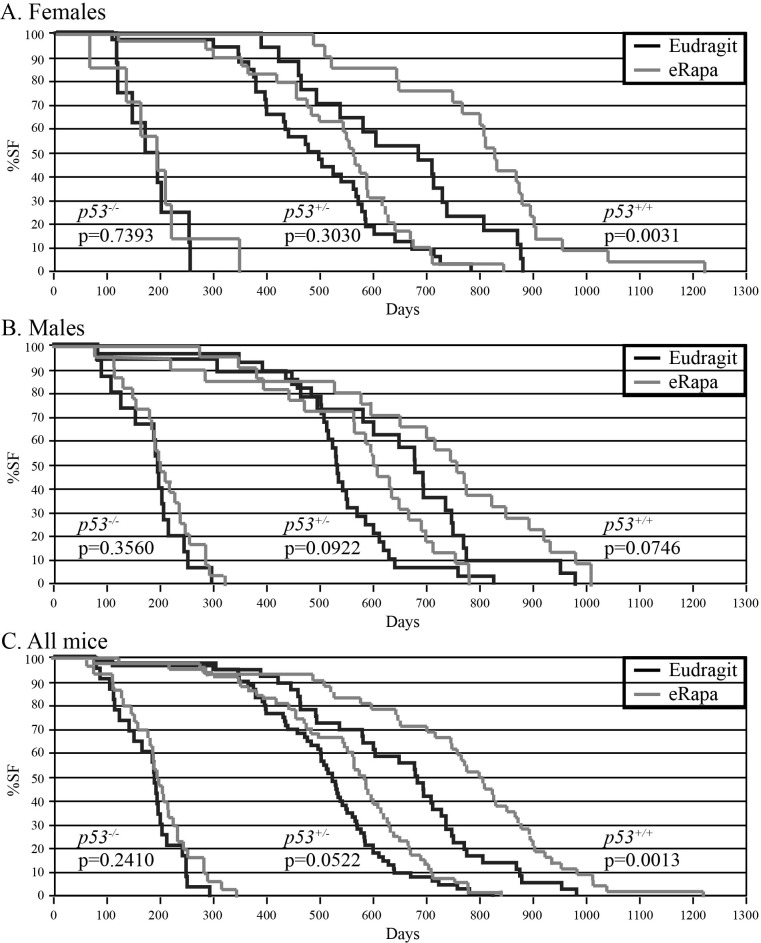
eRapa-mediated life span extension depends on p53 dose in mice Mice were fed eRapa (14 ppm) or empty capsules (Eudragit) starting at approximately 8.5 weeks of age until death. (**A**) Females. Number of Eudragit-fed mice: 8 *p53*^*−/−*^, 32 *p53*^*+/−*^, 17 *p53*^*+/+*^. Number of eRapa-fed mice: 7 *p53*^*−/−*^, 29 *p53*^*+/−*^, 21 *p53*^*+/+*^. (**B**) Males. Number of Eudragit-fed mice: 15 *p53*^*−/−*^, 28 *p53*^*+/−*^, 19 *p53*^*+/+*^. Number of eRapa-fed mice: 23 *p53*^*−/−*^, 22 *p53*^*+/−*^, 21 *p53*^*+/+*^. (**C**) Females and males combined. Number of Eudragit-fed mice: 23 *p53*^*−/−*^, 60 *p53*^*+/−*^, 36 *p53*^*+/+*^. Number of eRapa-fed mice: 30 *p53*^*−/−*^, 51 *p53*^*+/−*^, 42 *p53*^*+/+*^.

p53 genotype influenced rapamycin-mediated life span extension. eRapa did not significantly benefit *p53^−/−^* mice (Figure 1), which had median survival times (male + female) of 198.5 and 192 days for control- and eRapa-fed mice, respectively (p=0.2410). By contrast, eRapa extended the median survival of *p53^+/−^* (520 to 582 days, p=0.0522) and *p53^+/+^* mice (681 to 802.5 days, p=0.0013). eRapa improved median survival more for *p53^+/−^* females compared to *p53^+/−^* males (15.5% vs. 13.7% increase) and more for *p53^+/+^* females compared to *p53*^+/+^ males (20.9% vs. 11.6% increase), similar to previous reports [4, 35]. The p53 gene dose appears to directly correlate with rapamycin's effectiveness at extending mouse life span.

Chronic eRapa treatment did not significantly change tumor incidence in p53-deficient mice. Necropsies were performed on moribund mice after sacrifice or soon after natural death. Potential tumor masses and abnormal tissues were analyzed. Cancer, mostly lymphoma, was the major pathology identified at the time of death for all genotypes (Table 1). With control chow, *p53^−/−^* mice had a higher lymphoma incidence than *p53^+/−^* or *p53^+/+^* mice (70% vs. 59% and 53%, respectively). With eRapa chow, there was a lower trend in the incidence of lymphoma in *p53^−/−^* mice but corresponding higher trend in the incidence of sarcomas (including leiomyoma, rhabdomyosarcoma, hemangiosarcoma and osteosarcoma); there was a similar but less pronounced trend in *p53^+/−^* mice. Very little change was observed in wild type *p53^+/+^* mice, similar to a report that showed eRapa had only a minor effect on tumor multiplicity or burden in genetically heterogenous mice [35].

**Table 1:** Summary of histology

			Female			
	p53−/−		p53+/−		p53+/+	
observation	eudragit	eRapa	eudragit	eRapa	eudragit	eRapa
lymphoma	83.3% (5)	33.3% (2)	46.2% (6)	63.6% (7)	50% (4)	10% (1)
other cancer	16.7% (1)	66.7% (4)	23.1% (3)	9.1% (1)	0%	10% (1)
liver defects	0%	0%	30.8% (8)	27.3%	50% (4)	80% (8)
total cancer	100% (6)	100% (6)	69.2% (9)	72.7% (8)	50% (4)	20% (2)
total histo samples	6	6	13	11	8	10
total in cohort	8	7	32	29	17	21
			Male			
	p53−/−		p53+/−		p53+/+	
observation	eudragit	eRapa	eudragit	eRapa	eudragit	eRapa
lymphoma	64.3% (9)	44.4% (8)	71.4% (10)	20% (2)	57.1% (4)	70% (7)
other cancer	21.4% (3)	55.6% (10)	14.3% (2)	40% (4)	14.3% (1)	10% (1)
liver defects	14.3% (2)	0%	14.3% (2)	40% (4)	28.6% (2)	20% (2)
total cancer	85.7% (12)	100% (18)	85.7% (12)	60% (6)	71.4% (5)	80% (8)
total histo samples	14	18	14	10	7	10
total in cohort	15	23	28	22	19	21

Other cancers include sarcomas (leiomyo-, rhabdo-, hemangio-, and osteo-), hepatocellular carcinoma, carcinoma, primitive neuroectodermal tumor, malignant fibrous histiocytoma, and a few unidentified neoplasms. Liver defects include biliary duct hyperplasia, colangiohepatitis, hepatitis, cystic liver, lipidosis, necrosis, thrombus, and focal hepatocellular alteration. Low incidence of the following were also observed: extramedullary hematopoiesis/lymphoid hyperplasia, bladder cystitis with edema, nephritis, hydronephrosis, myocarditis, lung congestion, colitis, abdominal steatosis, and benign masses.

### p53 augments rapamycin-induced inhibition of SASP in normal human fibroblasts

We tested the possibility that p53 and rapamycin would have an additive effect at suppressing characteristics of cellular senescence, a process that limits cell growth [37]. Cellular senescence helps prevent the proliferation of potential cancer cells and is therefore, anti-oncogenic. Yet it also promotes the senescence-associated secretory phenotype (SASP) that can increase cancer risk. The SASP is a collection of secreted factors characteristic of senescent cells that accompanies growth arrest [38] and is conserved between human and mouse cells [39]. These factors include growth factors, proteases, cytokines and chemokines and together support cancer development and progression, and might also promote aging [40].

We examined the ability of p53 and rapamycin to influence the SASP in four different normal human diploid fibroblast cell strains. We assayed the levels of three different cytokines indicative of a SASP response (IL-6, IL-8 and IL-1α). For comparison we assayed levels of a cytokine that is not part of the SASP (IL-5). To study the effects of p53, we expressed in cells the genetic suppressor element 22 (GSE), a peptide that prevents p53 tetramerization and inactivates function [41]. Using this approach, we observed the impact of p53 on IR-induced geroconversion for cells exposed to rapamycin.

**Figure 2:**
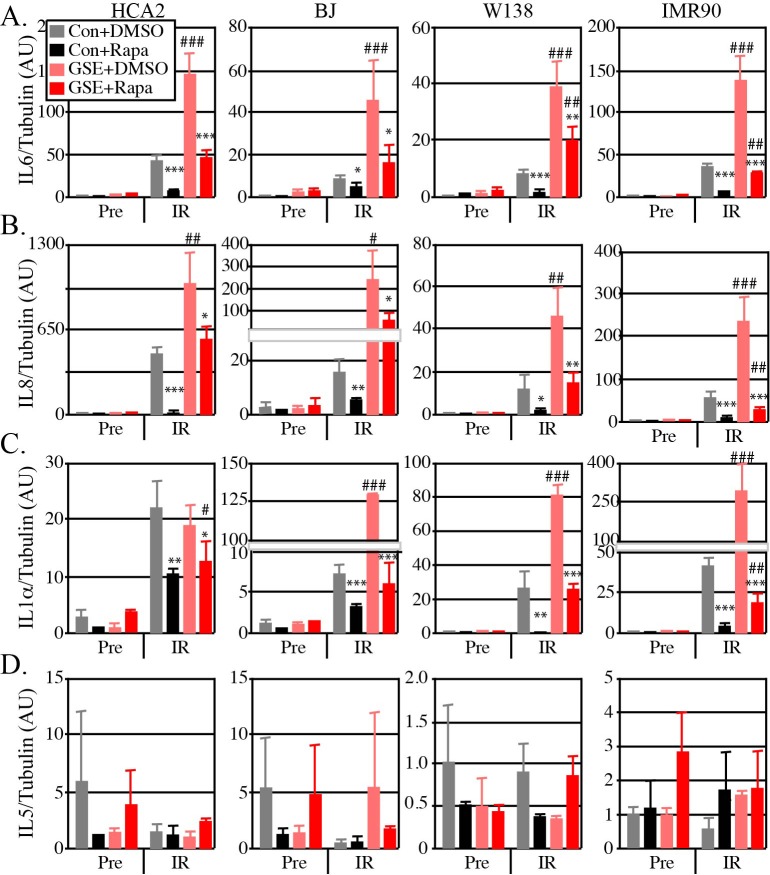
Rapamycin alters the SASP in normal human fibroblasts, and is more effective in cells with functional p53 Cells were infected with a lentiviral vector expressing GSE22 or an insertless vector. Irradiated and control cells were given Rapamycin (Rapa) or DMSO for 10 days. RNA was extracted from control and irradiated cells and quantified by qRT-PCR for mRNA levels of endogenous (**A**) IL6, (**B**) IL8, (**C**) IL1α and (**D**) IL5. Tubulin was used as internal control. Irradiated (IR) rapamycin treated cells compared to IR untreated cells N+4, data shown are the mean +/− SD: * p<0.05, ** p<0.01, ***<0.001. IR GSE cells compared to IR wild type cells: # p<0.05, ## p<0.01, ### p<0.001.

First we analyzed the impact of IR on these human cells. There was a very low level of expression of SASP-associated cytokines without IR exposure (Pre). As expected, IR increased the levels of SASP cytokines, but not IL-5, regardless of GSE22 expression (Figure 2, compare Pre vs IR). Thus, IR induced a SASP response as expected.

Second we analyzed the impact of p53 activity on the SASP. As expected [38], GSE22 significantly enhanced the SASP response for all cells and SASP cytokines (Figure 2, compare grey to pink bars) with a single possible exception (IL1α, HCA2 cells). Thus, p53 suppressed the SASP, consistent with the notion that p53 is a gerosuppressor under these conditions.

Third, we analyzed the impact of rapamycin on the SASP of irradiated cells without and with GSE22 expression. Rapamycin significantly suppressed the SASP for all cells, regardless of p53 status (Figure 2 compare grey vs black bars and pink vs red bars). Yet in every comparison, rapamycin suppressed the SASP to a greater extent in p53 competent cells (Figure 2, compare black and red bars). In most cases, rapamycin only decreased the SASP of GSE22-expressing cells to levels of irradiated untreated wild type cells. Thus, p53 augmented the effectiveness of rapamycin in reducing the SASP. This observation suggests p53 enables rapamycin and is consistent with the mouse life span studies.

### p53 augments rapamycin-induced elevation of amino acid levels in mouse ES cells

We tested the possibility that p53 and rapamycin would have an additive effect at altering cell metabolism since both the p53 and mTORC1 pathways are responsive to and influenced by the metabolic status of cells [3, 42]. Therefore, we measured metabolic parameters in *p53^+/+^* and *p53^−/−^* mouse ES cells [43] exposed to rapamycin. We chose ES cells because they are similar to cancer stem cells. Even though ES cells are primary, they are also pluripotent and immortal [44]. As such, ES cells exhibit self-renewal and are oncogenic. Furthermore, like many cancer cells, they have a high mitotic index and exhibit enhanced glycolysis (the Warburg effect) [45-48]. In ES cells, p53 activates genes associated with differentiation but suppresses genes associated with ES/iPS cell status in response to ionizing radiation [43]. Therefore, ES cells were used to assess the potential additive effect of rapamycin dose in *p53^+/+^* and *p53^−/−^* cells.

**Figure 3:**
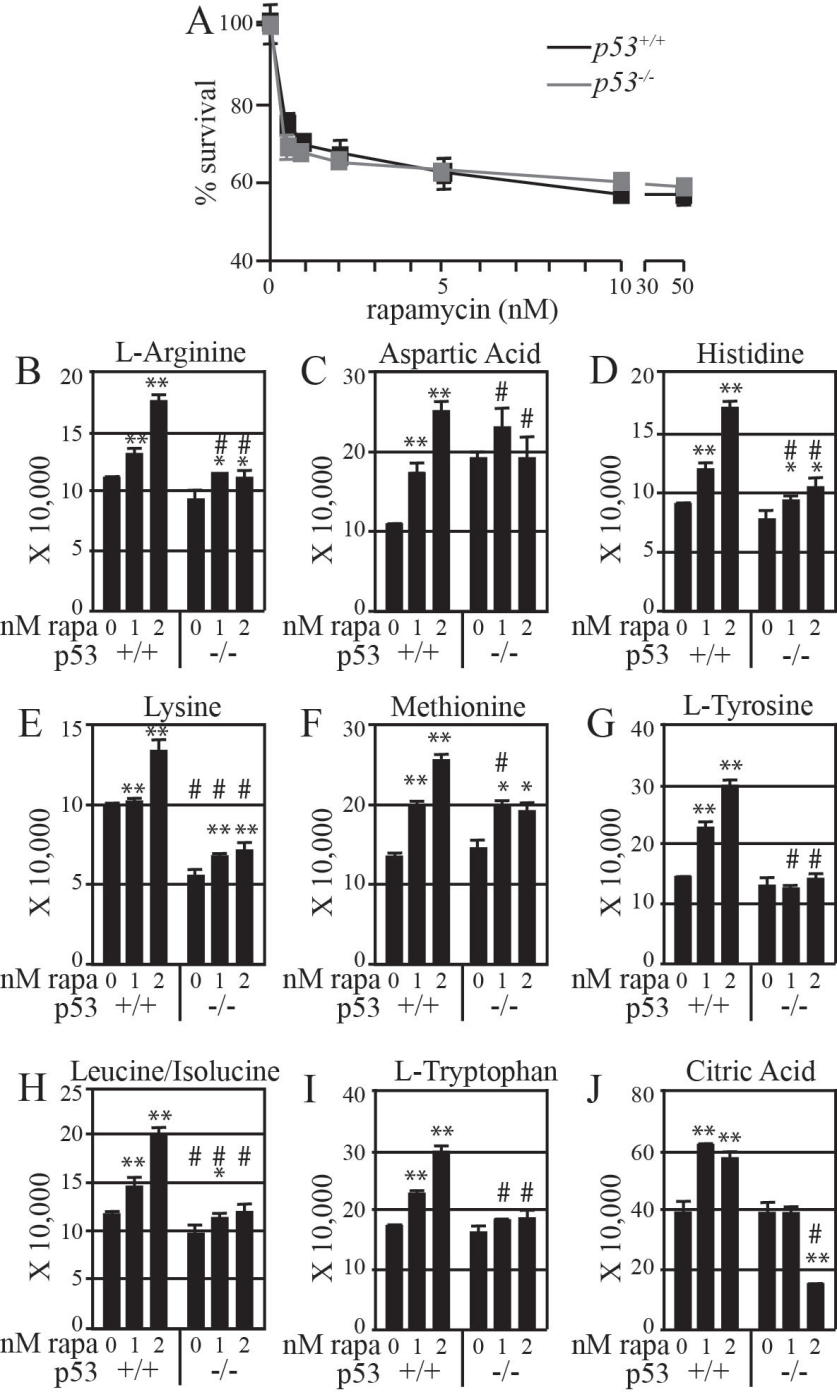
Rapamycin more effectively increases amino acid levels in mouse ES cells with functional p53 (**A**) Cell proliferation curve to find physiological rapamycin doses that are at the threshold concentration needed to impair cell proliferation. ES cells were treated for 48 hours with the indicated rapamycin concentrations (0-100 nM). Relative cell numbers were determined using MTT. In order to allow for the increased mitotic index in *p53*^*−/−*^ cells, values are plotted as a percentage of the control (0 nM rapamycin) for each cell type. (**B**-**I**) Amino acids. Note that rapamycin at 1 and 2 nM progressively increased the level of all amino acids. (**J**) Citric acid. Rapamycin exposed cells compared to unexposed wild type cells: * p<0.05, ** p<0.01. Mutant cell compared to wild type cells at a given rapamycin dose: # p<0.05.

Metabolic profiling was performed on mouse ES cells exposed to physiologically relevant doses of rapamycin, as determined by a proliferation curve that determined the effect of rapamycin on ES cell proliferation (Figure 3A). Rapamycin elevated the cellular levels of eight amino acids and p53 augmented this enhancement (Figure 3B-3I). mTOR signaling is responsive to amino acid levels, which are critical for protein translation [49, 50]. Rapamycin can elevate amino acid levels by elevating autophagy and promoting amino acid import by regulating expression of amino acid transporters such as Snat3 [51]. A similar result was seen for citric acid (Figure 3J). Citric acid is important to the tricarboxylic acid cycle, consistent with a role for p53 in promoting mitochondrial respiration [42, 52]. Thus, p53 augments rapamycin-mediated elevation of amino acid and citric acid levels in ES cells.

## DISCUSSION

We provide three lines of evidence that support the notion that p53 and rapamycin are additive. First, p53 enabled eRapa to extend life span in mice. Second, p53 facilitated the ability of rapamycin to suppress the IR-induced SASP in human cells. Third, p53 enabled rapamycin to elevate amino acid and citric acid levels in mouse ES cells. Thus, there appears to be an augmentative relationship between p53 and rapamycin.

**Figure 4:**
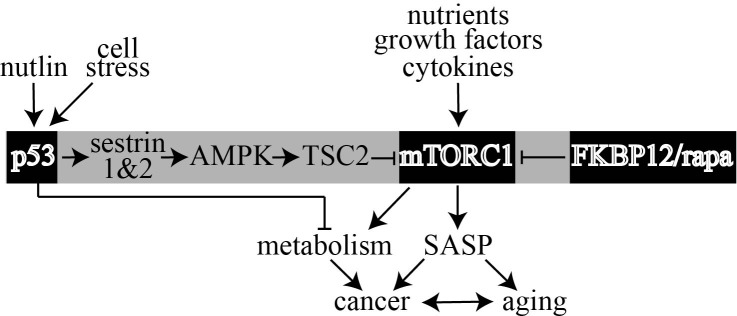
Model that shows rapamycin (rapa) and p53 separately antagonize mTORC1 through two distinct pathways to influence cancer, SASP and metabolism This model supports the possibility that p53 agonists like nutlin can enable rapamycin to further suppress mTORC1.

We suggest that p53 and rapamycin blunt mTORC1 activity through different pathways to result in this additive relationship (Figure 4). Previous publications support this possibility. Rapamycin/FKBP12 destabilizes the mTOR complex [1] while p53 induces transcription of Sestrin 1 and 2 to activate AMPK/TSC2 inhibition of mTOR [28]. Thus, our results are consistent with these separate modes of action to blunt mTORC1 since we find that p53 and rapamycin have an additive effect in three separate experiments. Even though p53 and rapamycin appear to suppress mTOR through distinct pathways, it still remains possible that rapamycin is partly dependent on p53 for its full effect on mTOR and therefore they are not strictly additive.

### p53 and rapamycin are integrated to affect mouse life span

Compared to wild type, mice deficient for p53 were cancer prone [23], whereas mice that overexpress p53 were cancer resistant [24]. Rapamycin could suppress oncogenesis to extend the life span of wild type mice. In support of this possibility, 14 ppm eRapa extended the life span of several cancer-prone mouse models with wild type p53 [8, 9]. In this study, 14 ppm eRapa extended the life span of *p53^+/+^* mice, and to a lesser degree *p53^+/−^* mice, but failed to extend the life span of *p53^−/−^* mice. By contrast, Rapatar (rapamycin in nanoformulated micelles) extended the life span of *p53^−/−^* mice (*p53^+/−^* and *p53^+/+^* mice were not reported) [11]. Mice exposed to Rapatar appeared to have higher serum levels than mice fed 14 ppm eRapa. For example, Rapatar exposure via IV or PO resulted in >100 ng/ml blood rapamycin for 4 hours after treatment or ~200 ng/ml blood rapamycin for 8 hours after treatment, respectively. By comparison, the mean blood rapamycin concentration for our mice fed 14 ppm eRapa was 2.57 +/− 1.98 ng/ml and the maximum concentration was 7.13 ng/ml. These values might be low since we analyzed only moribund mice but in another one of our studies using 14 ppm eRapa the blood concentration was typically 32 – 51 ng/ml for healthy mice [9]. Thus, increased rapamycin concentrations appeared to overcome a defect in p53 consistent with the notion that p53 and rapamycin are additive for extending mouse life span.

### p53 and rapamycin are integrated to affect SASP

p53 appears to facilitate two distinct biological outcomes that first induce, but then suppress cellular senescence [27]. The first biological outcome is a temporary cell cycle arrest (a reversible quiescent state) in response to stress [27, 53]. For example, p53 rapidly arrests cells in response to DNA damage and p53 is necessary for cellular senescence in DNA repair-defective cells [54]. Thus, the p53 mediated DNA damage response appears to promote cellular senescence in response to genotoxic stress [55]. However, this arrest is not senescence [56], but likely an intermediate step to senescence [57, 58]. While cells are arrested or in a quiescent state, p53 might actually inhibit cellular senescence [59, 60]. This possibility is consistent with our observation that p53 suppressed IR-induced SASP. p53 could suppress SASP by negatively regulating mTORC1 in response to stress [26, 27]. To support this possibility, mTORC1 drives p53-arrested cells into a permanent senescent state [61] and our data are consistent with these results since rapamycin augmented p53 suppression of the IR-induced SASP. The p53 + rapamycin-mediated reduction of SASP could suppress cancer [62]. A reduced SASP could also impact aging since the elimination of senescent cells ameliorated a variety of age-related pathologies in an early aging mouse model [63] and since eRapa ameliorated aging in wild type mice [4, 14-17]. Rapamycin also extended life span of lower organisms that do not develop cancer [19]. Thus, p53 induces biological outcomes both necessary (cell cycle arrest) and antagonistic (reduced SASP) to cellular senescence that could impact cancer and aging.

### p53 and rapamycin affect amino acid levels

Although the p53 DDR (growth arrest, senescence and apoptosis) has been extensively studied, recent results suggest that other p53 activities can suppress cancer, in particular those that affect cellular metabolism [64]. To that end, we performed metabolic profiling of *p53^+/+^* and *p53^−/−^* ES cells exposed to rapamycin. Previously, removal of amino acids was shown to suppress mTORC1, whereas an autophagy-mediated increase in amino acids was shown to reactivate mTORC1, thus, establishing a feedback loop [49, 65, 66]. We found that p53 enabled rapamycin to increase amino acid levels in ES cells. These results are consistent with the cancer-suppressive role of autophagy [67] and p53's influence on metabolism [42] with the general outcome of antagonizing mTORC1 activity and enhancing rapamycin effectiveness.

Autophagy also promotes recycling of damaged organelles in response to nutrient depletion, which is important for cell survival, and may be anti-aging owing to the degradation of damaged organelles [3]. The increase in amino acid levels promoted by rapamycin (and augmented by p53) might be the result of mTORC1 regulation of amino acid import via increased levels of transporters such as SNAT3 [51]. In this vein, p53 tumor suppression was shown to involve cell death via ferroptosis independent of its DNA damage response [68], which involved increased ROS that was controlled by expression of SLC7A11 (a cysteine/glutamate antiporter). Rapamycin-dependent alterations of citric acid levels might also be part of a similar cell survival response. Further unraveling the complex regulatory relationships between mTORC1 and p53 in cancer and aging will be an important goal of future studies.

### Therapeutic potential

Our results support the previous observations that p53 and rapamycin antagonize mTORC1 through separate modes of action. As such p53 and rapamycin appear to have an additive effect to inhibit mTORC1. This contention is relevant to the use of mTORC1 inhibitors [69] as anti-cancer therapeutics since p53 is mutated or dysfunctional in most cancers [32]. Taking this into account, mTORC1 inhibitors might be most effective early in the oncogenic process, before p53 is mutated. Our previous results support this possibility since we found that eRapa suppressed the development of tumors with wild type p53 [8, 9]. High levels of mTORC1 inhibitor might be needed to overcome cancer with p53-null mutations. Yet for those p53-dysfunctional cancers that have a functional p53 protein, mTORC1 inhibitors coupled with p53 enablers such as nutlin [53] could be a powerful combination allowing a lower dose for each (Figure 4).

## MATERIALS AND METHODS

### Mice and lifespan determination

All mouse procedures were carried out in accordance with The Guide for the Care and Use of Laboratory Animals and approved by the institutional IACUC. *p53^+/−^* mice [23] were bred in-house to generate the *p53^+/+^*, *p53^+/−^* and *p53^−/−^* littermates for testing eRapa. Littermates or closely age-matched animals of the same sex were housed together regardless of genotype. Genotyping was done as previously described [23]. The mice were allowed to live their normal lifespan and were sacrificed only when moribund (immobile and unable to reach the water bottle).

### Rapamycin and control diets

Mice were started on control chow or chow containing microencapsulated rapamycin at approximately 8.5 weeks of age (median age 61 days for eudragit and rapamycin with a range of 45-73 days for eudragit and 45-72 days for eRapa). Rapamycin chow contained microencapsulated rapamycin at a concentration of 14 mg/kg food (14 ppm), which provides a dose of approximately 2.24 mg of rapamycin per kg of body weight. The control diet was identical but with empty capsules. Diets were prepared by TestDiet, Inc. (Richmond, IN), using Purina 5LG6 as the base [4].

### Rapamycin blood levels

Measurement of rapamycin levels in the blood was performed by HPLC-tandem MS as previously described [4]. Blood was obtained from moribund mice prior to sacrifice when possible.

### Pathology

Fixed tissues (in 10% neutralized formalin) were embedded in paraffin, sectioned at 5 μm and stained with hematoxylin-eosin. Diagnosis was determined as previously described by the Pathology core of the Barshop Institute for Longevity and Aging Studies [70-73].

### Cell culture and proliferation assays

Primary human fibroblasts HCA2, BJ, WI38 and IMR90 were cultured in DMEM supplemented with 10% FBS (Corning, CellGro). The GSE22 peptide was expressed and used to inhibit p53 function as previously described [41]. Cells were treated with 12.5 nmol of rapamycin (Sigma R0395) or equal volume of DMSO for 10 days and media were refreshed every 2 days. Four replicates were assayed for each cell type and each condition. Mouse *p53^+/+^* and *p53^−/−^* embryonic stem cells were cultured on gelatinized plates without feeder cells, as previously described [74, 75]. For cell proliferation assays, cells were plated at 1×10^4^ cells per well in 24-well plates. After 24 hr, fresh medium with rapamycin (Sigma R0395) at the indicated concentrations was added to each well. Cells were allowed to grow for an additional 48 hr before harvest; relative cell number was determined using the MTT assay [76]. Six replicates were assayed for each rapamycin concentration.

### SASP quantification

Human primary fibroblasts were exposed to 10 Gy X-ray or mock irradiation, as previously described [38]. Irradiated cells were cultured for 10 days and control cells for 48 hours. Cells were harvested and RNA was extracted. cDNA was synthesized and analyzed by qPCR using the UPL system (Roche), as previously described [77]. Primers and probes were as follow:

IL-6: #45 FW 5′-gcccagctatgaactccttct-3′;RV 5′-gaaggcagcaggcaacac-3′

IL-8: #72 FW 5′-agacagcagagcacacaagc-3′;RV 5′-atggttccttccggtggt-3′

IL-1α: #6 FW 5′-ggttgagtttaagccaatcca-3′;RV 5′-tgctgacctaggcttgatga-3′

IL-5: #6 FW 5′-cactgaagaaatctttcagggaat-3′;RV 5′-ccgtctttcttctccacacttt-3′

### Metabolic profiling

*p53^+/+^* and *p53^−/−^* cells [43] were grown in 10 cm plates and treated with 0, 1 or 2nM rapamycin for 24 hours before harvest. Cell number, viability and size were determined using the Countess Automated Cell Counter (Invitrogen). Equal numbers of cells were used for each assay; 6 independent plates were assayed for each cell type and condition. The cells were extracted with ice-cold 80% aqueous methanol and maintained at −20 °C for 1 h. Subsequently, the extracts were centrifuged at 13,800 × g for 10 minutes and the supernatants were transferred for HPLC electrospray ionization-mass spectrometry (HPLC-ESI-MS) analysis. HPLC-ESI-MS analyses were conducted on a Thermo Fisher Q Exactive mass spectrometer with on-line separation by a Thermo Fisher/Dionex Ultimate 3000 HPLC. HPLC conditions were: column, Luna NH2, 3 μm, 2 × 150 mm (Phenomenex); mobile phase A, 5% acetonitrile in water containing 20 mM ammonium acetate and 20 mM ammonium hydroxide, pH 9.45; mobile phase B, acetonitrile; flow rate, 400 μL/min; gradient, 85% B to 1% B over 10 minutes and held at 1% B for 10 minutes. Data-dependent MS/MS scans were performed with one full scan followed by 6 MS/MS scans in the HCD collision cell with normalized collision energy (NCE) of 35 arbitrary units. Both positive and negative ion detection were performed through at 70,000 resolution (m/z 300). Progenesis CoMet (Nonlinear Dynamics) was used to process the raw data files to detect the metabolites that exhibit significant differences in the intensity among the different groups. Peak alignment and integration was performed and the relative abundance was generated for each metabolite among different sample types. The metabolites were identified with accurate mass through Metlin databases searching using a 5-ppm mass tolerance and manual interpretation of the MS/MS fragment patterns. The comparison with the retention times with commercially available standards was also performed for further confirmation.
